# Shifts in *Borrelia burgdorferi* (*s.l.*) geno-species infections in *Ixodes ricinus* over a 10-year surveillance period in the city of Hanover (Germany) and *Borrelia miyamotoi*-specific Reverse Line Blot detection

**DOI:** 10.1186/s13071-018-2882-9

**Published:** 2018-05-18

**Authors:** Katrin Blazejak, Marie-Kristin Raulf, Elisabeth Janecek, Daniela Jordan, Volker Fingerle, Christina Strube

**Affiliations:** 1 Institute for Parasitology, Centre for Infection Medicine, University of Veterinary Medicine Hannover, Buenteweg 17, 30559 Hanover, Germany; 2 Immunology Unit and Research Center for Emerging Infections and Zoonoses, University of Veterinary Medicine Hannover, Buenteweg 17, 30559 Hanover, Germany; 3 German National Reference Centre for Borrelia, Oberschleißheim, Germany

**Keywords:** Lyme disease, Tick-borne diseases, Ticks, *Borrelia*, Rickettsiales, *Borrelia miyamotoi*

## Abstract

**Background:**

Lyme borreliosis caused by spirochetes of the *Borrelia burgdorferi* (*sensu lato*) complex is still the most common tick-borne disease in Europe, posing a considerable threat to public health. The predominant vector in Europe is the widespread hard tick *Ixodes ricinus*, which also transmits the relapsing fever spirochete *B. miyamotoi* as well as pathogenic Rickettsiales (*Anaplasma phagocytophilum*, *Rickettsia* spp.). To assess the public health risk, a long-term monitoring of tick infection rates with the named pathogens is indispensable.

**Methods:**

The present study is the first German 10-year follow-up monitoring of tick infections with *Borrelia* spp. and co-infections with Rickettsiales. Furthermore, a specific Reverse Line Blot (RLB) protocol for detection of *B. miyamotoi* and simultaneous differentiation of *B. burgdorferi* (*s.l.*) geno-species was established.

**Results:**

Overall, 24.0% (505/2100) of ticks collected in the city of Hanover were infected with *Borrelia*. In detail, 35.4% (203/573) of adult ticks [38.5% females (111/288) and 32.3% males (92/285)] and 19.8% nymphs (302/1527) were infected, representing consistent infection rates over the 10-year monitoring period. Geno-species differentiation using RLB determined *B. miyamotoi* in 8.9% (45/505) of positive ticks. Furthermore, a significant decrease in *B. afzelii* and *B. spielmanii* infection rates from 2010 to 2015 was observed. Co-infections with *Rickettsia* spp. and *A. phagocytophilum* increased between 2010 and 2015 (7.3 *vs* 10.9% and 0.3 *vs* 1.1%, respectively).

**Conclusions:**

Long-term monitoring is an essential part of public health risk assessment to capture data on pathogen occurrence over time. Such data will reveal shifts in pathogen geno-species distribution and help to answer the question whether or not climate change influences tick-borne pathogens.

## Background

Lyme borreliosis (LB) is still the most common tick-borne disease in the Northern Hemisphere and poses a considerable risk to public health [1]. Estimated LB incidences in Europe total approximately 85,000 cases annually [2]. Data from mandatory notifications from six federal states in the eastern part of Germany show that the overall incidence varied between 34.9 cases/100,000 inhabitants in 2009 and 19.54 cases/100,000 inhabitants in 2012 [3]; extrapolation to the total German population results in approximately 15,000 to 30,000 LB cases per year. The resulting impact on public health care systems is substantial due to annual medical costs, which are estimated for Germany alone to be 23.7 million Euros for LB hospital care and 51.2 million Euros for outpatients. Additionally, indirect costs due to a loss of productivity are estimated at 7.1 million Euros annually [4]. In Europe, *B. burgdorferi* (*sensu lato*) spirochetes are mostly transmitted by their main vector, the castor bean tick *Ixodes ricinus* [1]. To date, 22 geno-species of the *B. burgdorferi* (*s.l.*) complex have been identified, with 11 of them occurring throughout Europe [5].

In addition, *B. miyamotoi*, belonging to the relapsing fever (RF) borreliae, has been detected in *I. ricinus* in Europe, which also represents their main vector as opposed to related RF borreliae [6–8]. *Borrelia burgdorferi* (*s.l.*) prevalences in ticks show large differences nationwide [9–18] with exemplarily 3.1% *Borrelia-*positive ticks at the German Baltic coast [13] and considerably higher infection rates of 34.1% in the northern German city of Hamburg [12] or 36.2% in the southern federal state Bavaria [14]. Conversely, *B. miyamotoi* tick infection rates vary between 1.8% [18] and 2.4% in western as well as 2.7% in southern Germany [19], whereas data from northern Germany are not yet available. In humans, *B. miyamotoi* mostly causes influenza-like illnesses with fever, nausea, fatigue, chills, headache or myalgia [6, 20], resulting in challenging diagnostics as symptoms are rather unspecific. However, severe cases of *B. miyamotoi*-induced meningoencephalitis have been described in immunocompromised patients [21, 22].

Here, we present long-term data in terms of a 10-year follow-up monitoring on tick infection rates with *Borrelia* spp. and co-infections with Rickettsiales in the city of Hanover, Germany, to contribute to public health risk assessment.

## Methods

### Tick material and sampling sites

From April to October 2015, a total of 2100 questing ticks were collected by the flagging method at ten different recreation areas evenly distributed in the city of Hanover as described previously [23]. The locations comprised extensive forest areas as well as inner urban parks, frequently visited by inhabitants and tourists. The study design was based on previous studies conducted in 2005 and 2010 [9–11] and served simultaneously as a 10-year follow-up monitoring. A total of 30 ticks per month was collected at the same sampling locations previously examined in 2010 and 2005 [9–11]. However, due to construction measures, two sampling sites differed between 2015 and 2010 compared to 2005.

Tick species and developmental stage identification of collected ticks was performed according to morphological keys described by Estrada-Peña et al. [24]. Ticks were individually stored at -20 °C until genomic DNA isolation.

### Genomic DNA isolation and detection of *Borrelia* spp. by quantitative real-time PCR

Ticks were individually homogenized and genomic DNA isolation was performed as described elsewhere [23, 25, 26]. Isolated DNA was stored at -20 °C until further use. A qPCR targeting the *5S*-*23S* rRNA intergenic spacer (IGS) based on a primer-probe combination designed by Strube et al. [11] was performed as described previously [9, 12].

### Design of a *B. miyamotoi*-specific Reverse Line Blot (RLB) probe

For detection of *B. miyamotoi* by Reverse Line Blot (RLB), a species-specific probe was designed based on seven published sequences targeting the hydrolase-23S rRNA IGS of *B. miyamotoi*. Sequences were retrieved from the GenBank database (National Centre for Biotechnology Information) under accession numbers GQ387038 (Switzerland), CP017126, AY531879, CP010308, CP006647 (USA) and CP004217 (Japan). Furthermore, *B. miyamotoi* (strain HT 31) DNA was amplified by RLB-PCR using primers B-5SBor and 23SBor [27] to obtain a 400 bp fragment of the hydrolase-23S rRNA IGS. The PCR product was cloned and sequenced to identify candidate regions for probe design. Therefore, the obtained sequence of *B. miyamotoi* strain HT 31 was aligned with named sequences (GenBank: GQ387038, CP017126, AY531879, CP010308, CP006647 and CP004217) using the Clone Manager Professional software (version 9, Scientific and Educational Software, Denver, USA).

### Reverse Line Blot (RLB)

To determine geno-species of *Borrelia* spp. positive tick samples, the RLB technique was performed as described previously [9, 12] with few modifications. RLB-PCR included biotin-linked B5S-Bor forward and 23S-Bor reverse primer as described by Alekseev et al. [27] to amplify the *B. burgdorferi* (*s.l.*) *5S*-*23S* rRNA IGS target region, which occurs as duplicate in the genome of the *B. burgdorferi* (*s.l.*) complex. For amplification of *B. miyamotoi*, a hydrolase-23S rRNA region specific, biotin-linked forward primer was designed and included in the reaction mixture (BMiya-For: 5'-TTA GGA TTA ATG ATR TTK TTA CC-3'). The 25 μl reaction mixture contained 1 μl B5S-Bor forward primer and 1 μl BMiya-For in addition to 2 μl 23S-Bor reverse primer (10 μM each), 0.5 μl deoxynucleoside triphosphates (10 mM each), 2.5 μl 10× Taq buffer and 0.125 μl (5 U/μl) Taq DNA polymerase (Qiagen, Hilden, Germany). Ten microlitres of tick DNA sample or 1 μl (0.1 ng/μl) control *Borrelia* DNA was utilized and the amount of double-distilled water adjusted accordingly. Controls comprised 14 *Borrelia* strains, which were included in each run: *B. afzelii* (strain PBas), *B. garinii* (PWudII), *B. bavariensis* (Pbi), *B. bissettiae* (DN127), *B. burgdorferi* (*s.s.*) (PAbe), *B. lusitaniae* (PotiB2), *B. spielmanii* (PHap), *B. valaisiana* (VS116), *B. carolinensis*, *B. kurtenbachii* (25015), *B. miyamotoi* (HT31), *B. hermsii* (DSM4682), *B. recurrentis* and *B. duttonii*. Evaluation of MIYA probe additionally included *Treponema phagedenis* and *Leptospira* spp. DNA to exclude cross-reaction with related spirochetes. PCR cycling conditions included an initial activation step at 94 °C for 3 min, followed by 45 cycles of 94 °C for 20 s, 52 °C for 30 s, 72 °C for 30 s and a final elongation step at 72 °C for 7 min. Subsequently, 20 μl of tick PCR products as well as 10 μl of positive controls and a no-template control were hybridized to 8 different oligonucleotide probes linked to the membrane as described previously [9, 10] with the following modifications of probe concentrations: *B. burgdorferi* (*s.l.*) (SL2): 0.67 μM [9]; *B. afzelii* (AF): 0.03 μM [28]; *B. garinii* (GA): 0.67 μM [28]; *B. bissettiae* (BISNE2): 0.67 μM [29]; *B. burgdorferi* (*s.s.*) (SS): 0.67 μM [29]; *B. lusitaniae* (LUSINE2): 0.67 μM [29]; *B. spielmanii* (SpiNE3): 6.7 μM [30]; *B. valaisiana* (VSNE): 0.67 μM [30]; and *B. miyamotoi* (MIYA): 0.67 μM (this study).

Chemiluminescent signals were detected using Amersham™ ECL™ Prime Western Blotting Detection Reagent (GE Healthcare Life Sciences, Freiburg, Germany) according to the manufacturer’s instructions, followed by signal detection for a duration of 30 s to 3 min light exposition using Chemiluminescent-Imager Celvin®S 320+ (Biostep, Jahnsdorf, Germany).

As the *B. garinii* (GA) probe detects both *B. garinii* and *B. bavariensis*, Sanger sequencing of positive samples was performed to differentiate between these geno-species as described previously [9, 12]. Similarly, the *B. burgdorferi* (*s.s.*) (SS) probe detects *B. burgdorferi* (*s.s.*) and *B. carolinensis*. On some blots weak signals of the *B. bissettiae* (BISNE2) probe with *B. kurtenbachii* were also observed. Thus, samples showing a positive RLB signal for these probes were also sent for custom Sanger sequencing.

### Statistical analyses

Data were statistically analysed by the Chi-square test with subsequent Bonferroni-Holm correction or Fisher’s exact test, depending on sample sizes, as carried out previously [9, 10]. Data analyses included comparison of infection rates between stages (total ticks, adult females, adult males, nymphs), sampling months and locations. Furthermore, comparison among stages, locations and seasons between 2015 and 2010 as well as 2005 was performed. As sampling locations, months and number of collected ticks were identical in the years 2010 and 2015 and nearly identical in 2005 (two locations differed due to construction measures), a direct comparison of obtained data between study years was feasible. For comparison of stage-related infection rates, data obtained in 2005 was included as reported by Tappe et al. [9]. To enable data comparison of the seasonal distribution over the 10-year sampling period, data obtained in 2010 and 2005 were modified according to data obtained in 2005 [31]. As the sampling periods in 2010 and 2015 started in April, instead of March/April as in 2005 [31], statistical analyses were not feasible for these sampling months. Due to variations of two sampling sites between 2005 and the follow-up studies conducted in 2010 and 2015, comparison of local distribution was not feasible for two sampling sites (“Mecklenheide” and “Seelhorster Wald”) between 2005 *vs* 2010 and 2015. All analyses were performed with the GraphPad Prism™ software (version 6.03, La Jolla, CA, USA).

## Results

### Tick material

Based on macroscopic identifiable morphologic parameters, all collected ticks were classified as *I. ricinus.* Identification of the developmental stage resulted in 573 adults (288 females and 285 males) and 1527 nymphs.

### *Borrelia* infections in ticks in 2015

In 2015, a total of 24.1% (505/2100) of ticks were infected with *Borrelia* spp., subdivided into 35.4% adults (203/573), thereof 38.5% females (111/288) and 32.3% males (92/285), as well as 19.8% nymphs (302/1527). Infection rates of different stages resulted in significant differences between nymphs *vs* females as well as males (*χ*^2^ = 21.34–47.48, *df* = 1, *P* < 0.0001).

Concerning seasonal variations, no significant differences were detected between individual sampling months. Conversely, tick infection rates varied significantly between different sampling sites. The highest infection rate of 31.4% (66/210) was determined in the inner urban park “Georgengarten” which differed significantly from sampling sites “Annateiche” (14.8%, 31/210; *χ*^2^ = 15.50, *df* = 1 *P* < 0.0001) and “Ricklinger Teiche” (15.2%, 32/210; *χ*^2^ = 14.49, *df* = 1 *P* = 0.0001), which showed the lowest determined tick infection rates. Detailed information about seasonal distribution of *Borrelia* infected tick stages is provided in Table 1, information about local distribution is provided in Table 2.

**Table 1:** Seasonal distribution of *Borrelia* spp. infected Hanoverian ticks (positives/total ticks) in 2015

Seasonal distribution	April	May	June	July	August	September	October	Total
Adults	27/95	33/97	42/95	25/80	29/75	28/72	19/59	203/573
(%)	(28.4)	(34.0)	(44.2)	(31.3)	(38.7)	(38.9)	(32.2)	(35.4)^a^
Adult males	13/53	12/48	21/51	10/42	18/37	11/28	7/27	92/285
(%)	(24.5)	(25.0)	(41.2)	(23.8)	(48.6)	(39.3)	(25.9)	(32.3)^a^
Adult females	14/43	21/49	21/44	15/38	11/38	17/44	12/32	111/288
(%)	(32.6)	(42.9)	(47.7)	(39.5)	(28.9)	(38.6)	(37.5)	(38.5)^a^
Nymphs	49/205	39/203	52/205	40/220	32/225	48/228	41/241	302/1527
(%)	(23.9)	(19.2)	(25.4)	(18.2)	(14.2)	(21.1)	(17.0)	(19.7)^a^
Total	76/300	72/300	94/300	65/300	61/300	76/300	61/300	505/2100
(%)	(25.3)	(24.0)	(31.3)	(21.7)	(20.3)	(25.3)	(20.3)	(24.1)

^a^Significantly higher infection rates in adults (females and males) *vs* nymphs (*P* < 0.05)

**Table 2:** Local distribution of *Borrelia* spp. infected Hanoverian ticks (positives/total ticks) in 2015

Local distribution	Mecklenheide	Große Heide	Misburger Wald	Annateiche	Seelhorster Wald	Ricklinger Teiche	Bornumer Holz	Georgengarten	Eilenriede	Maschpark
Adults	27/72	39/84	6/20	7/40	36/93	13/40	27/74	14/36	17/91	7/23
(%)	(37.5)	(46.2)	(30.0)	(17.5)	(38.7)	(32.5)	(36.5)	(38.9)	(18.7)	(30.4)
Adult males	13/35	14/37	1/11	2/21	24/45	5/23	14/39	6/19	11/46	2/9
(%)	(37.1)	(37.8)	(9.1)	(9.5)	(53.3)	(21.7)	(35.9)	(31.6)	(23.9)	(22.2)
Adult females	14/37	25/47	5/9	5/19	12/48	8/17	13/35	8/17	16/45	5/14
(%)	(37.8)	(53.2)	(55.6)	(26.3)	(25.0)	(47.1)	(37.1)	(47.1)	(35.6)	(35.7)
Nymphs	34/138	24/126	43/190	24/170	20/117	19/170	25/136	52/174	14/119	47/187
(%)	(24.6)	(19.0)	(22.6)	(14.1)	(17.1)	(11.2)	(18.4)	(29.9)	(11.8)	(25.1)
Total	61/210	63/210	49/210	31/210	56/210	32/210	52/210	66/210	41/210	54/210
(%)	(29.0)b	(30.0)b	(23.3)	(14.8)b	(26.7)	(15.2)b	(24.8)	(31.4)b	(19.5)	(25.7)

^b^Significantly higher infection rates in “Mecklenheide”, “Große Heide” and “Georgengarten” *vs* “Annateiche” and “Ricklinger Teiche”(*P* < 0.05)

### Tick infections with *Borrelia* in 2015 *vs* 2010 and 2005

**Fig. 1:**
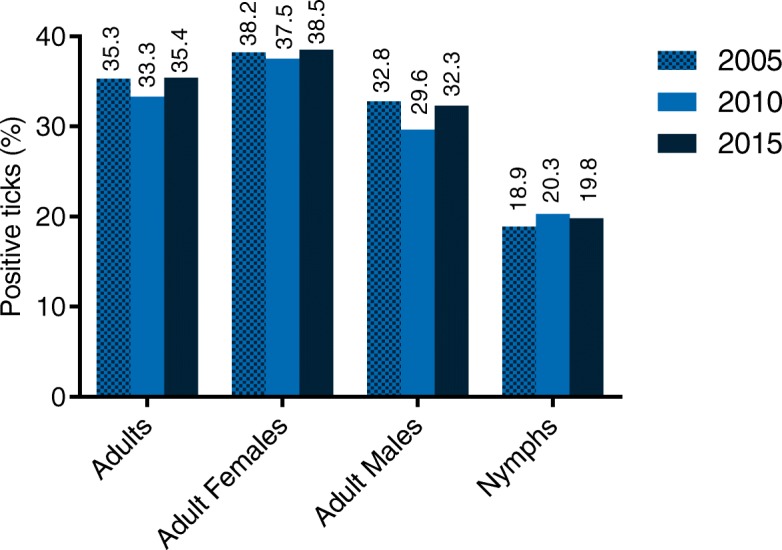
tick infection rates with *Borrelia* spp. over the ten-year monitoring period (2005 [11] *vs* 2010 [9] *vs* 2015)

**Fig. 2:**
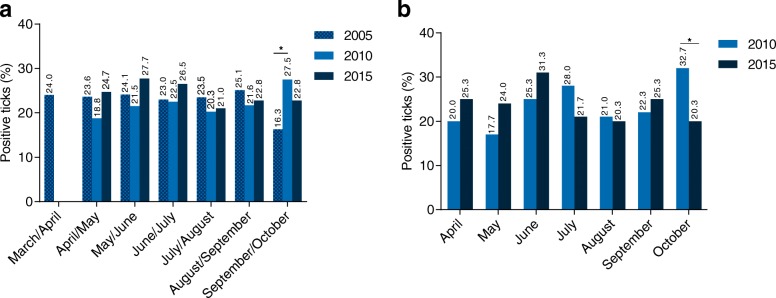
seasonal distribution of *Borrelia-*positive ticks in **a** 2005–2015 (seasonal data of individual sampling months was not obtained in 2005, wherefore data obtained in 2010 and 2015 was modified accordingly) [9, 31] and **b** 2010 [9] *vs* 2015. *P ≤ 0.05

Over the entire monitoring period of ten years, total tick infection rates as well as infection rates in different stages remained consistent (Fig. 1), which was also observed for different sampling sites when comparing 2010 [9] to 2015. Concerning local infection rates, a significant decrease was determined at “Ricklinger Teiche” between 2005 and 2015 (31.7 *vs* 15.2%; *χ*^2^ = 17.30, *df* = 1, *P* < 0.0001). Contrary, infection rates significantly increased between 2005 and 2015 at “Georgengarten” (19.3 *vs* 31.4%; *χ*^2^ = 10.06, *df* = 1, *P* = 0.0015) as well as “Maschpark” (14.3 *vs* 27.7%; *χ*^2^ = 7.565, *df* = 1 *P* = 0.0059). Furthermore, significantly higher *Borrelia* infection rates (*χ*^2^ =11.09, *df* = 1, *P* = 0.0009) were determined in October 2010 (32.7%) [9] than in October 2015 (20.3%). Comparison of seasonal data over the ten-year period revealed significantly higher infection rates in September/October 2010 [9] than in September/October 2005 [31] (27.5 *vs* 16.3%; *χ*^2^ = 9.244, *df* = 1, *P* = 0.0024). Detailed comparison of the seasonal distribution in 2005, 2010 and 2015 is depicted in Fig. 2.

### Design of the *Borrelia miyamotoi* probe MIYA

**Fig. 3:**
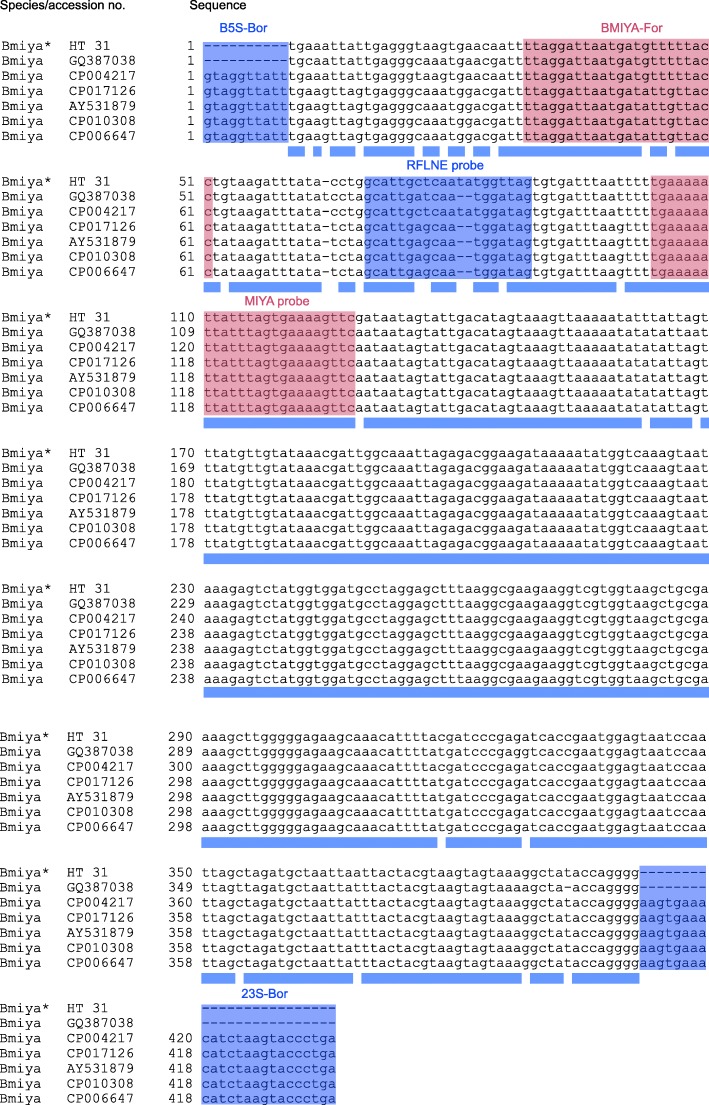
Alignment of the hydrolase-23S rRNA IGS sequence of *Borrelia miyamotoi* from different origins. Substitutions and insertions/deletions are located to the loci of the RFLNE probe (blue) [28], whereas probe MIYA (red) displays 100% identity with aligned sequences

**Fig. 4:**
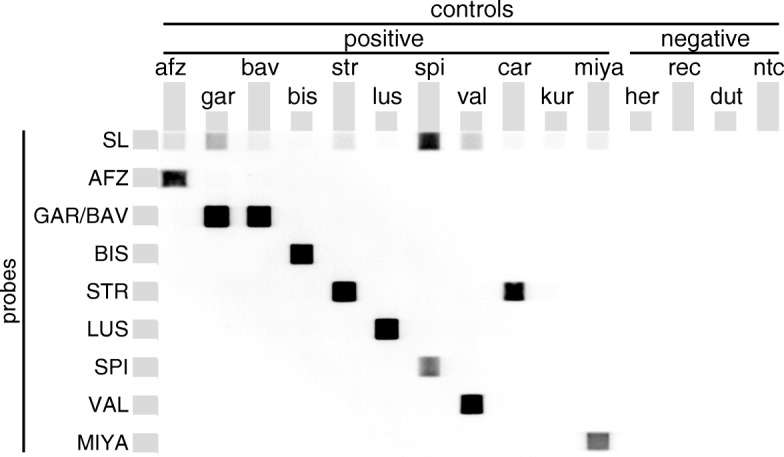
Exemplary Reverse Line Blot (RLB) image, showing the specific detection of amplified *B. miyamotoi* (miya) DNA by the newly designed probe MIYA. Additionally, detection of *B. burgdorferi* (*s.l.*) geno-species by different probes (SL, *B. burgdorferi* (*s.l.*); AFZ, *B. afzelii*; GAR/BAV, *B. garinii*/*B. bavariensis*; BIS, *B. bissettiae*; STR, *B. burgdorferi* (*s.s.*); LUS, *B. lusitaniae*; SPI, *B. spielmanii*; VAL, *B. valaisiana*) is shown. Controls were DNA amplification products of *B. afzelii* (afz), *B. garinii* (gar), *B. bavariensis* (bav), *B. bissettiae* (bis), *B. burgdorferi* (*s.s.*) (str), *B. lusitaniae* (lus), *B. spielmanii* (spi), *B. valaisiana* (val), *B. carolinensis* (car), *B. kurtenbachii* (kur), *B. hermsii* (her), *B. recurrentis* (rec) and *B. duttonii* (dut). *Abbreviation*: ntc, no-template control

The obtained sequence of *B. miyamotoi* strain HT 31 showed 99% identity with *B. miyamotoi* from Japan and 95% identity with sequences from the USA. The newly designed probe MIYA targeting the hydrolase-23S rRNA IGS showed 100% sequence identity with all available sequences contrary to the previously used RFLNE probe [9, 10, 12] designed by Gern et al. [29] (Fig. 3). Comparison of the 25 bp probe MIYA with published sequences of the hydrolase-23S IGS region of other relapsing fever *Borrelia* and the 5S-23S IGS region of *B. burgdorferi* (*s.l.*) as well as related species showed no similarities to respective loci (data not shown). Additionally, no cross-reactions with DNA from different geno-species of the *B. burgdorferi* (*s.l.*) complex, the relapsing fever borreliae *B. duttonii*, *B. hermsii* and *B. recurrentis* (Fig. 4) as well as *Treponema phagedenis* and *Leptospira* spp. (data not shown) were observed.

### *Borrelia* geno-species identification in ticks

*Borrelia* geno-species differentiation by RLB was successful in 52.7% (266/505) of qPCR *Borrelia-*positive ticks. Sensitivity of conducted RLB was dependent on the number of *5S*-*23S* IGS copies, resulting in 100% (15/15) differentiation of samples containing ≥ 10^4^ copies, 91.7% (55/60) of samples containing ≥ 10^3^ copies, 84.6% (121/143) of samples containing ≥ 10^2^ copies and 50.4% (57/113) of samples containing ≥ 10^1^ copies. Of samples containing ≤ 10 copies, only 10.3% (18/174) were differentiated. *Borrelia afzelii* was the most frequently occurring geno-species (16.8%; 85/505) followed by *B. garinii*/*B. bavariensis* as well as *B. valaisiana* (each 10.7%; 54/505) *B. burgdorferi* (*s.s.*) (6.5%; 33/505), *B. spielmanii* (3.0%; 15/505), *B. bissettiae* (1.6%; 8/505) and *B. lusitaniae* (0.4%; 2/505). The relapsing fever species *B. miyamotoi* was detected in 8.9% (45/505) of *Borrelia*-positive ticks. Regarding multiple-infections, the majority of ticks (46.5%; 235/505) were mono-infected, 5.5% (28/505) were double-infected and 0.6% (3/505) were triple-infected.

Out of 54 *B. garinii*/*B. bavariensis* RLB positive tick samples, 43 were successfully identified by Sanger sequencing. Of these, 97.7% (42/43) were identified as *B. garinii* and 2.3% (1/42) as *B. bavariensis*. Out of the 33 *B. burgdorferi* (*s.s.*)/*B. carolinensis* RLB-positive tick samples, 26 (78.8%) were identified by Sanger sequencing. Of these all (26/26) were identified as *B. burgdorferi* (*s.s.*) and none (0/26) as *B. carolinensis*. Out of the 8 *B. bissettiae*/*B. kurtenbachii* RLB-positive tick samples, none (0/8) were successfully identified by Sanger sequencing. Detailed information on geno-species distribution in *Borrelia*-positive ticks is given in Table 3.

**Table 3:** Reverse Line Blot results on *B. burgdorferi* (*s.l.*) geno-species and *B. miyamotoi* distribution in infected (*n* = 505) ticks in 2015

Total no. of infections	No. of infected ticks (%)	Mono-infection	No. of infected ticks (%)	Double-infection	No. of infected ticks (%)	Triple-infection	No. of infected ticks (%)
*Baf*	85 (16.8)	*Baf*	70 (13.9)	*Baf* + *Bss*/*Bcar*	7 (1.4)	*Baf* + *Bbi*/*Bku* + *Bss*/*Bca*	3 (0.6)
*Bga*/*Bba*	54 (10.7)	*Bva*	47 (9.3)	*Bga*/*Bba + Bva*	6 (1.2)		
*Bva*	54 (10.7)	*Bga*/*Bba*	46 (9.1)	*Baf + Bga*/*Bba*	1 (0.2)		
*Bss*/*Bca*	33 (6.5)	*Bss*/*Bca*	19 (3.8)	*Bga*/*Bba* + *Blu*	1 (0.2)		
*Bsp*	15 (3.0)	*Bsp*	13 (2.6)	*Baf + Bsp*	1 (0.2)		
*Bbi*/*Bku*	8 (1.6)	*Blu*	1 (0.2)	*Baf* + *Bmi*	3 (0.6)		
*Blu*	2 (0.4)	*Bbi*/*Bku*	0 (0.0)	*Bsp* + *Bmi*	1 (0.2)		
*Bmi*	45 (8.9)	*Bmi*	39 (7.7)	*Bva* + *Bmi*	1 (0.2)		
No geno-species determined	239 (47.3)			*Bss*/*Bca* + *Bmi*	1 (0.2)		

*Abbreviations*: *Baf*, *B. afzelii*; *Bva*, *B. valaisiana*; *Bss*, *B. burgdorferi* (*s.s*.); *Bca*, *B. carolinensis*; *Bga*, *B. garinii*; *Bba*, *B. bavariensis*; *Bsp*, *B. spielmanii*; *Blu*, *B. lusitaniae*; *Bbi*, *B. bissettiae*; *Bku*, *B. kurtenbachii*; *Bmi*, *B. miyamotoi*

### *Borrelia* geno-species in 2015 *vs* 2010 and 2005

The number of tick mono-infections with *B. afzelii*, and *B. spielmanii* decreased significantly from 2010 [9, 10] to 2015 (*B. afzelii*: 30.9 *vs* 16.8%, *χ*^2^ = 26.02, *df* = 1, *P* < 0.0001; *B. spielmanii*: 10.9 *vs* 3.0%, *χ*^2^ = 23.13, *df* = 1, *P* < 0.0001). In the study year 2015, *B. afzelii* was the predominant geno-species, followed by *B. garinii*/*B. bavariensis* and *B. valaisiana* (each 10.7%, see Table 3). In 2010, *B. afzelii* as the predominant geno-species was followed by *B. garinii*/*B. bavariensis* and *B. spielmanii* [9, 10], whereas in 2005, *B. garinii*/*B. bavariensis* was the predominant geno-species, followed by *B. afzelii* and *B. spielmanii* [11]. A significant decrease in double-infections between 2015 *vs* 2010 (5.5% *vs* 13.4%, *χ*^2^ = 17.08, *df* = 1, *P* < 0.0001) [9, 10] and 2005 (24.3%, *χ*^2^ = 55.01, *df* = 1, *P* < 0.0001) [11] was determined, whereas numbers of triple-infections remained consistent between years. Furthermore, no quadruple-infection was determined in 2015 contrary to 2010 [9, 10].

### Tick co-infections with *Borrelia* and Rickettsiales in 2015 *vs* 2010 and 2005

In 2015, 10.9% (229/2100) of ticks were co-infected with *Rickettsia* species, whereas co-infections with *A. phagocytophilum* were detected in 1.1% (23/2100) of ticks. Furthermore, 0.6% (12/2100) of ticks were infected with all three pathogens (Table 4). Significantly increased numbers of *Borrelia*/*Rickettsia* spp. (10.9 *vs* 7.3%; *χ*^2^ = 16.20, *df* = 1, *P* < 0.0001) as well as *Borrelia*/*A. phagocytophilum* (1.1 *vs* 0.3%; *χ*^2^ = 7.554, *df* = 1, *P* = 0.006) co-infected ticks were detected in 2015 *vs* 2010 [9, 10], whereas consistent co-infection rates were found between 2015 *vs* 2005. Concerning triple-infections, no significant differences were found between study years.

**Table 4:** Coinfections with *B. burgdorferi* (*s.l.*) and Rickettsiales in Hanoverian *I. ricinus* in 2015

	No. of collected ticks	No. of *B. burgdorferi* (*s.l.*) positive ticks	Total coinfections	*Rickettsia* spp. coinfections	*A. phagocytophilum* coinfections	Coinfections with *Rickettsia* spp. and *A. phagocytophilum*
		No. (%)	No. (%)	No. (%)	No. (%)	No. (%)
Adult	573	203 (35.4)	113 (20.0)	92 (16.1)	14 (2.4)	7 (1.22)
Males	285	92 (32.3)	49 (17.2)	40 (14.0)	5 (1.8)	4 (1.4)
Females	288	111 (38.5)	64 (22.2)	52 (18.1)	9 (3.1)	3 (1.0)
Nymphs	1527	302 (19.8)	151 (9.8)	137 (9.0)	9 (0.6)	5 (0.33)
All stages	2100	505 (24.1)	264 (12.5)	229 (10.9)	23 (1.1)	12 (0.6)

## Discussion

Besides Lyme borreliae of the *B. burgdorferi* (*s.l.*) complex, the relapsing fever agent *B. miyamotoi* has frequently been detected in *I. ricinus* in Europe during the last decade and human pathogenicity has been observed [6, 32]. As both pathogens utilize *I. ricinus* as vector, monitoring of tick infection rates is an indispensable part of a public health risk assessment. To date, no data about *B. miyamotoi* tick infection rates in northern Germany are available wherefore an RLB-protocol was established, enabling specific detection of *B. miyamotoi* in addition to *B. burgdorferi* (*s.l.*) geno-species differentiation. By comparing current data with those obtained in 2010 and 2005, the first 10-year follow-up monitoring of *Borrelia* tick infections in Germany was performed. Over this period, infection rates were consistent in all investigated tick stages, but varied at three sampling sites. Furthermore, geno-species distribution showed significant differences. As certain geno-species are reliant on specific reservoir hosts (e.g. rodents or birds), observed variations may reflect alterations in the availability of reservoir host species between study years.

In 2015, approximately one quarter of examined ticks was infected with *Borrelia* spp., with adult ticks being significantly more often infected than nymphs. This was also observed in the studies in 2005 and 2010 and is explainable by efficient transstadial transmission [33] as well as an increased probability to acquire spirochetes by an extra blood meal taken during the nymphal stage [9]. Local tick infection also differed significantly within the city of Hanover in 2015, ranging from a minimum of 14.8% at “Annateiche”, an extensive forest area, to a maximum of 31.4% at “Georgengarten”, an inner urban public park. As high *Borrelia* prevalences were also found at other sampling sites characterized by extensive forest areas, respective infection rates are most likely explainable by local abundance of infected reservoir hosts. Local abundance of reservoir hosts could possibly be influenced by landscape features and high frequency of visitors, as the increase in infection rates between 2005 and 2015 was only observed for urban parks. Presumably, intense usage and cultivation of landscape areas may lead to an increased density of e.g. bank voles or other small rodents, serving as suitable reservoir hosts. In contrast, wildlife fauna in forest areas may be more diverse and include species such as roe deer or wild boar which are unsuitable *Borrelia* hosts, possibly leading to a dilution effect [34]. Varying tick infection rates at individual sampling sites over the ten-year monitoring period again indicate an altered distribution of *Borrelia* spp. infections in reservoir hosts [33]. Differences in (micro)climatic conditions as well as the local vegetation between study years probably contribute to the altered annual *Borrelia* spp. infection rates by affecting both, reservoir host and questing tick abundance.

Contrary to local differences in 2015, *Borrelia* spp. infection rates showed no seasonal variations. When comparing seasonal data of 2015 *vs* 2010, significantly decreased infection rates were found for October 2015. Climatic differences, like a possibly more moderate and humid autumn in 2010, may account for this difference between the years, since they may have an impact on reservoir host availability besides tick density and activity [1].

*Borrelia* geno-species differentiation by RLB was successful in 52.7% of infected ticks. Compared to the study in 2010 [9, 10], the RLB procedure in 2015 was modified to enable successful *B. miyamotoi* amplification. RLB hybridisation (Fig. 4) showed that available probes for differentiation of *B. burgdorferi* (*s.l.*) geno-species generally recognise all geno-species occurring in Europe (however, subsequent sequencing is needed to differentiate some geno-species due to cross-reactions) with the limitation that detection of *B. finlandensis* was not tested as no positive control strain could be included. Even though no specific RLB-probe for *B. finlandensis* is available, it seems unlikely that undetermined tick samples were infected with that particular geno-species as it was found in only one *I. ricinus* in Europe previously [35]. Undetermined samples rather result from the lower RLB sensitivity compared to qPCR, which detects one target copy in the reaction set-up [9, 11], whereas the RLB identified the infecting geno-species in only 10.3% of samples containing ≤ 10 *5S*-*23S* rRNA IGS copies in the recent study. Despite consistent total *Borrelia* infection rates, RLB revealed variations in distribution of different *B. burgdorferi* (*s.l.*) geno-species between 2015 and 2010. Interestingly, a significant decrease in the rodent associated geno-species *B. afzelii* and *B. spielmanii* [36, 37] was determined in 2015 *vs* 2010. The decrease may be based on a correlation between available vertebrate reservoir hosts and distribution of *Borrelia* species in ticks [37, 38]. Such correlations may also explain higher *B. garinii* infection rates in 2005 and 2015 *vs* 2010 [9–11]. Generally, it should be noted that geno-species differentiation in 2005 was achieved by conventional PCR instead of RLB as performed in 2010 [9] and in the current study. Regarding multiple-infections, double-infections significantly decreased in 2015 compared to 2010 [9]. In 2010, double-infections were mainly caused by *B. afzelii* and *B. spielmanii* [9], and decreased occurrence of named geno-species in 2015 may have consequently resulted in decreased numbers of total double-infections. *B. afzelii* was found in all determined triple-infections in the present study and was frequently found in triple-infections in 2010 [9]. However, triple-infections appear to be a consistently rare event as ticks are required to feed from multiple hosts, multiple-infected hosts or acquire an infection *via* co-feeding with (multiple-) infected ticks [5, 39]. The absence of quadruple-infections in 2015 was also not unexpected, as detection was only described once in 2010 [9] but not in 2005 [11].

A total of 8.9% (45/505) *Borrelia-*positive ticks were infected with *B. miyamotoi*, representing the first available prevalence data for northern Germany. The developed *B. miyamotoi* specific RLB-probe presumably enables detection of worldwide occurring *B. miyamotoi* strains (Fig. 3) and is therefore highly valuable. As *B. miyamotoi* may cause mild as well as severe disease in humans [6, 21, 22, 40], the occurrence in ticks in inner urban recreation areas should be considered as a risk to public health and monitored accordingly, in particular as 8.9% of ticks identified for their *Borrelia* geno-species were infected with this pathogen. Even though the presence of *B. miyamotoi* was not investigated in previous studies conducted in Hannover, the detection of *Borrelia-*positive *I. ricinus* larvae in 2010 [9] indicate its occurrence due to highly efficient transovarial transmission in ticks contrary to inefficient transovarial transmission of *B. burgdorferi* (*s.l.*) [9, 41]. Furthermore, successful transmission of *B. miyamotoi* from infected *I. ricinus* larvae to vertebrates has been shown in *in vivo* experiments, indicating an infection risk from larval tick bites [42]. Therefore, further examination of larval *I. ricinus* populations would aid in assessing the risk of transmission to humans. In this context, it is essential to sensitise clinicians to consider *B. miyamotoi* as a causative agent when treating patients with unspecific feverish infections as *B. miyamotoi* induces rather unspecific symptoms or may mimic other tick-borne diseases like human granulocytic anaplasmosis (HGA) [43]. Mimicking HGA may occur frequently as about 14.6% of patients with suspected but serologically unconfirmed HGA were determined anti-*B. miyamotoi* antibody positive in the Netherlands [44].

Concerning co-infections with Rickettsiales, increased co-infection rates with *Rickettsia* spp. in 2015 may above all be based on generally increased *Rickettsia* spp. infection rates in 2015 *vs* 2010 (50.8 *vs* 26.2%) [23, 25, 26]. Co-infections of *Borrelia* spp. with *A. phagocytophilum* also increased from 2010 to 2015, but not from 2005 to 2015, although tick infection rates of both pathogens remained consistent [17, 23, 25, 26]. Further monitoring regarding co-infection rates are desired as investigations of experimentally *A. phagocytophilum*/*B. burgdorferi* (*s.s.*) co-infected mice showed increased bacterial load and pathogen transmission to the tick vector as well as an increase in severity of Lyme disease compared to mono-infections [45, 46]. Therefore, even though numbers of *Borrelia*/*A. phagocytophilum* co-infected ticks are generally low, potentially enhanced severity in Lyme disease manifestation should increase awareness of pathogen transmission from co-infected ticks.

## Conclusions

The RLB protocol established in the present study enables specific detection of *B. miyamotoi* besides differentiation of common European *B. burgdorferi* (*s.l.*) geno-species, resulting in the first detection of *B. miyamotoi* in *I. ricinus* in northern Germany. Although the overall determined *Borrelia* tick infection rate remained consistent over the 10-year monitoring period, shifts in occurrence of human pathogenic geno-species underline the importance of detailed analyses for diagnostic and research purposes as well as for a public health risk assessment.

## Abbreviations

RLB: Reverse line blot
